# Neuro-ophthalmic Afferent System Diagnoses A General Ophthalmologist Should (Almost) Never Make Alone

**DOI:** 10.4103/0974-9233.48867

**Published:** 2009

**Authors:** Andrew G. Lee

**Affiliations:** From the Departments of Ophthalmology, Neurology, and Neurosurgery, the H. Stanley Thompson Neuro-ophthalmology Clinic at the University of Iowa Hospitals and Clinics, Iowa City, Iowa, United States of America

**Keywords:** Optic Atrophy, Posterior Ischemic Optic Neuropathy, Chronic Optic Neuritis, Papilledema

## Abstract

The general ophthalmologist might be called upon to make the diagnosis of neuro-ophthlamic conditions which are either rare or require extensive testing to exclude alternative diagnoses. This paper reviews some common afferent system neuro-ophthlamologic diagnoses that the general ophthalmologist should rarely if ever make alone. These include posterior ischemic optic neuropathy, chronic optic neuritis, retinal migraine, and optic atrophy. Although these diagnoses do exist they are typically diagnoses of exclusion that require neuroimaging and ancillary testing and have no diagnostic test to confirm the diagnosis.

Comprehensive ophthalmologists are on the front lines of general eye care and may encounter specific neuro-ophthalmic diagnoses which although they do exist require caution and extensive testing to confirm the diagnosis. In this article we will cover neuro-ophthalmic afferent system diagnoses that a general ophthalmologist should almost never make alone because of the dangers of misdiagnosis. These include: 1) unexplained optic atrophy, 2) posterior ischemic optic neuropathy, 3) chronic optic neuritis, 4) papilledema, and 5) ocular migraine in elderly patients. These diagnoses share common characteristics that make them particularly dangerous. First these diagnoses really do exist although they are rare. Second, they tend to have no specific diagnostic test to establish the diagnosis and are typically considered to be “diagnoses of exclusion”. Third, alternative etiologies for the clinical presentations are potentially dangerous and misdiagnosis can lead to visual loss, morbidity or mortality. Fourth, cases may have “negative” imaging and generalists should insure that the findings resolve or remain stable with serial follow up. Finally, if there is progression or other atypical features of the case the comprehensive ophthalmologist should consider referring the patient to a neuro-ophthalmologist.

Part of the problem for the busy comprehensive ophthalmologist is that for much of our work day the clinical findings and the final diagnoses are one and the same. For example, in a typical day an ophthalmologist might diagnose a posterior subcapsular cataract, rhegmatogenous retinal detachment, or a central retinal vein or central retinal artery occlusion. The slit lamp or ophthalmoscopic findings are essentially the same as the final diagnosis. Unfortunately, in neuro-ophthalmic conditions the clinical finding is not the same as the final diagnosis. For example the findings of an optic neuropathy or optic atrophy are not diagnoses per se and require further evaluation.

## CASE ONE: OPTIC ATROPHY IS NOT A DIAGNOSIS

A 55-year-old man with hypertension and diabetes presents with new awareness of blurred vision OD for two months. The visual acuity was 20/25 OD and 20/20 OS. Goldmann perimetry demonstrated an inferior altitudinal visual field loss OD. The pupil examination showed a relative afferent pupillary defect (RAPD) OD. Ophthalmoscopy showed a pale optic nerve OD. The eye exam was normal OS. A diagnosis of “optic atrophy” OD was made and the patient was instructed to return in 3 months for a repeat visual field. The patient returns in 3 months and the vision is now counting fingers OD. Magnetic resonance imaging of the head and orbit showed optic nerve sheath enhancement OD consistent with an optic nerve sheath meningioma.

Optic atrophy is not a diagnosis. The clinician should document a cause for the optic atrophy and if no etiology is established a neuroimaging study and other evaluation should be performed. In a 55-year-old vasculopathic male the most likely etiology for a unilateral optic neuropathy with optic atrophy is non-arteritic anterior ischemic optic neuropathy (NA-AION). The “anterior” in NA-AION refers to optic disc edema and the absence of documented disc edema phase should prompt consideration for evaluation for alternative etiologies. In this case the patient experienced acute awareness of his visual loss OD rather than acute onset and the disc edema phase was not seen. Although serial visual field and follow up for a presumptive diagnosis of optic atrophy due to NA-AION is reasonable the clinical plan should be well documented in the chart, the patient should be informed of the possibility for a compressive or other etiology, and imaging and other evaluation should be pursued if the patient has progressive signs or symptoms (as in this case).

Lee et al. previously reported on the diagnostic yield for the evaluation of isolated and unexplained optic atrophy. This study was a retrospective review of all charts with the diagnosis of optic atrophy. Included patients were adults with isolated, but unexplained, optic atrophy and patients were excluded if they were children, had incomplete or inadequate documentation of the findings, had non-neurologically isolated optic atrophy (e.g., other localizing findings), or had a history (e.g., prior neuroimaging study showed a compressive lesion, prior ischemic optic neuropathy) or examination (e.g., central retinal artery occlusion) evidence for an etiology for the optic atrophy. A total of 1110 charts with the diagnosis of optic atrophy were reviewed from the two participating institutions (368 from the University of Cincinnati and 742 from the University of Iowa). Of these 1110 charts, 91 (8%) with isolated unexplained optic atrophy were included, and 1019 charts (92%) were excluded. Of the 91 cases included 18 (20%) had an underlying compressive lesion causing optic atrophy and 73 (80%) cases had no etiology for the optic atrophy on neuroimaging. Of the 18 patients with abnormal neuroimaging studies (e.g., meningioma, pituitary adenoma, or craniopharyngioma) 11 had bilateral and 7 had unilateral optic atrophy. Five of the 18 patients had progressive visual loss, 3 had hemianopic visual field loss, and 11 were younger than 50 years old. This study demonstrates that the risk of missing a compressive lesion in unexplained optic atrophy is high (20%) and that clinicians should not make the diagnosis of optic atrophy alone without an underlying proven or postulated mechanism.

## CASE TWO: POSTERIOR ISCHEMIC OPTIC NEUROPATHY (PION) IS A DIAGNOSIS OF EXCLUSION

A 65-year-old woman with hypertension and diabetes presents with new awareness of blurred vision OD for two days. The visual acuity was 20/40 OD and 20/20 OS. No visual field is performed. The pupil examination showed a relative afferent pupillary defect (RAPD) OD. Ophthalmoscopy showed a normal optic nerve OD. The remainder of the eye exam was normal OS. A diagnosis of “posterior ischemic optic neuropathy” (PION) is made and the patient is instructed to return for a “next available” visual field test. The patient returns two months later and reports that “his vision is worse”. The vision is now 20/400 OD and 20/25 OS. There is a right RAPD. The Goldmann visual field showed a bitemporal hemianopic visual field loss with a superimposed central loss OD. There is now optic atrophy OD. A cranial MRI showed a pituitary adenoma compressing the optic nerve OD. A new eye doctor sees the patient and tells her that “If only you'd come to me sooner you would not be blind.”

The diagnosis of posterior ischemic optic neuropathy (PION) is one of exclusion. PION does occur and can be classified as arteritic (i.e., related to giant cell arteritis) or non-arteritic. The non-arteritic form is generally seen in the setting of perioperative or post-operative ischemic optic neuropathy after spine or cardiovascular surgery. Post-operative PION however has been seen after multiple surgical procedures.

Sadda et al. reported a retrospective chart review of 72 patients (98 eyes) with a clinical diagnosis of PION: perioperative (28 patients), arteritic (6 patients), and nonarteritic systemic vascular disease (38 patients). Among eyes with nonarteritic PION, 34% experienced improvement in vision, 28% remained stable, and 38% worsened.

It is my opinion that before the diagnosis of PION can be made two conditions must be excluded. First giant cell arteritis and second a compressive lesion producing a retrobulbar optic neuropathy. The key feature differentiating AION from PION is the presence of optic disc edema in the acute setting. Retrobulbar optic neuritis is typically a disorder of young patients and most commonly associated with multiple sclerosis. Retrobulbar optic neuritis however is rare in the elderly and the diagnosis should not be made without an imaging study. Compressive lesions can mimic an acute onset as seen in ischemic disease. In addition, corticosteroids a common treatment of retrobulbar optic neuritis can be disastrous in a patient harboring a fungal orbital apex lesion producing the retrobulbar optic neuropathy. Thus the diagnosis of retrobulbar “optic neuritis” should be made with extreme caution in the elderly. I generally recommend that a fluorescein angiogram be considered in elderly patients with suspected PION. The finding of choroidal perfusion deficit in the setting of PION is strongly suggestive of giant cell arteritis. Corticosteroids might be useful empirically for both arteritic and nonarteritic PION but an imaging study as mentioned above might be advisable prior to starting treatment.

I would accept the diagnosis of PION of the clinical findings are consistent with an optic neuropathy (i.e., visual loss, RAPD, optic neuropathy related visual field), there is a clear etiology (i.e., post-surgical PION or giant cell arteritis), and optic atrophy develops over time. If there is no clear etiology for the PION, then a neuroimaging study is recommended (preferably cranial MRI with contrast and orbital fat suppressed views).

## CASE THREE: CHRONIC OPTIC NEURITIS IS A DANGEROUS DIAGNOSIS

A 25-year-old woman presents with chronic and unresolved loss of vision OD that occurred acutely two months prior to presentation. There is a right RAPD, a central scotoma OD on visual field testing, and a normal optic disc. Computed tomography (CT) scan of the head in the emergency room was normal. A diagnosis of chronic optic neuritis” is made.

Chronic “optic neuritis” is a real diagnosis but the clinician should be careful to insure that the patient has a confirmed diagnosis of multiple sclerosis and no alternative etiology for the optic neuropathy. Typical optic neuritis resolves (20/40 or better vision) in over 90% of cases and lack of improvement should be a big “red flag” that the diagnosis is incorrect. In my experience patients with chronic”“optic neuritis” due to demyelinating disease often have a systemic MS course that mirrors the course of their optic neuropathy (i.e., primary or secondary progressive rather than relapsing-remitting course). One should also be cautious of using the term “optic neuritis” when one truly means optic neuropathy. Misusing or mixing terms (e.g., papilledema) or creating neologisms (e.g., “ischemic optic neuritis”) also is potentially misleading to your colleagues. The bottom line is that typical optic neuritis usually gets better over time and patients with an atypical course (e.g., “chronic optic neuritis”) should have evaluation for alternative etiologies for their optic neuropathy including neuroimaging. There are potentially dangerous conditions that could mimic the presentation of acute painful optic neuropathy and suggest a diagnosis of demyelinating optic neuritis (e.g., pituitary apoplexy or ophthalmic artery aneurysm). In addition there are subsets of variants of demyelinating optic neuropathy that may not have as favorable a rate of recovery and therefore might suggest alternative diagnoses (e.g., neuromyelitis optica). Finally, some patients with the mitochondrial disorder, Leber hereditary optic neuropathy (bilateral acute visual loss with cecocentral scotomas in a young male) can be associated with MS like illness. Although rare, I would accept the diagnosis of “chronic optic neuritis” as a diagnosis of exclusion if a high quality MR scan of the head and orbit with gadolinium has excluded a compressive lesion, the patient has clear diagnosis of prior multiple sclerosis, and alternative MS mimics have been considered and adequately excluded. I typically will inform the treating neurologist of the findings and my concerns and if there is any history of transverse myelitis or if the optic neuropathy is bilateral then I would consider evaluation for neuromyelitis optica (NMO) including NMO antibody testing. I would also consider ordering the mitochondrial genetic testing for Leber hereditary optic neuropathy if the patient has bilateral central or cecocentral scotomas especially in a young man.

## CASE FOUR (A): PAPILLEDEMA: WHAT'S IN A NAME?

A 30-year-old man presents with acute onset of unilateral optic disc edema with a macular star figure of exudate OD. The vision was 20/200 OD, there is right RAPD, and a cecocentral scotoma on visual field testing OD. There is mild vitreous cell OD. The ophthalmologist writes'“papilledema” on the chart and refers the patient to a neurologist for further testing. The neurologist orders a head MRI which is normal and performs a lumbar puncture that shows a normal opening pressure and cerebrospinal fluid analysis. The patient sees another ophthalmologist for a second opinion and the correct diagnosis of cat scratch neuroretinitis is made based upon a markedly elevated Bartonella henselae titer (IgG 1:1024).

Papilledema for neuro-ophthalmologist represents a term that is best reserved for optic disc swelling due to elevated intracranial pressure. It is my recommendation that all other causes of optic disc swelling should be referred to by etiology (e.g., optic neuritis, or anterior ischemic optic neuropathy) or simply by describing the ophthalmoscopic finding of “optic disc edema” Clinicians should use the term “papilledema” carefully because it is potentially confusing to your colleagues in neurology, neurosurgery, and medicine if you misuse the term. This inappropriate charting could lead to inappropriate testing? (e.g., the MRI and lumbar puncture in this case). It also delays testing for the real culprit for the patient's optic disc edema and a macular star figure (i.e., infectious cat scratch neuroretinitis in this case). The misleading documentation could also lead to medicolegal implications of the missed diagnosis.

In general the clinician can use the clinical presentation to differentiate the three major forms of optic disc edema: papilledema, anterior ischemic optic neuropathy, and optic neuritis. Papilledema can occur at any age, is typically associated with relatively preserved central visual acuity (unless there is macular exudate, fluid, hemorrhage, or chronic or long standing papilledema), is bilateral (although markedly asymmetric or frankly unilateral cases do occur), and usually produces a big blind spot initially on visual field testing followed by nerve fiber layer loss over time. There might also be associated signs and symptoms of increased intracranial pressure (e.g., headache, nausea, vomiting, pulse synchronous tinnitus, or diplopia from a nonlocalizing sixth nerve palsy). In contrast to papilledema, optic neuritis typically affects younger patients, is often associated with decreased central acuity and dyschromatopsia, is typically unilateral (although bilateral cases can occur especially in children), and the visual field defect is usually a central scotoma (although any type of optic nerve related visual field loss may occur). Finally, in an older adult, anterior ischemic optic neuropathy is the most common unilateral cause for optic disc edema. The patient may have vasculopathic risk factors, an altitudinal visual field defect (but any optic nerve type of defect can occur), and again is usually unilateral. Thus the clinician should be wary of making the diagnosis of bilateral simultaneous optic disc edema related to either optic neuritis or non-artertic anterior ischemic optic neuropathy and probably should exclude papilledema in these clinical settings.

The evaluation of papilledema should include a check of the systemic blood pressure as malignant hypertension can mimic papilledema (i.e., hypertensive optic disc edema). Neuroimaging (preferably an MRI with contrast and an MR venogram to exclude venous sinus thrombosis) should be considered as the initial study in papilledema. If the neuroimaging study is normal then a lumbar puncture with an opening pressure and cerebrospinal fluid analysis should be performed. If the intracranial pressure is elevated, the neuorimaging is normal, and the CSF analysis is normal then the diagnosis of idiopathic intracranial hypertension can be confirmed but should be considered a diagnosis of exclusion. Although a CT scan might be the initial emergent neuroimaging study performed, there are many etiologies for increased ICP that may be missed on CT scan especially a noncontrast study in the emergency room. These intracranial etiologies include cerebral venous sinus occlusion, arteriovenous or dural malformation, infiltrative neoplasm or disorders that produce meningeal enhancement (e.g., inflammatory, infectious, infiltrative, or neoplastic etiologies).

Although routine, typical, and medication responsive papilledema from idiopathic intracranial hypertension might be managed by a general ophthalmologist, a referral to neuro-ophthalmology might still be necessary if the patient fails maximum medical therapy or presents with acute, severe visual loss. These patients might require surgical intervention (e.g., shunting procedure or optic nerve sheath fenestration) to prevent irreversible papilledema related visual loss. In addition, the diagnosis should be made with caution in the following atypical circumstances: the patient is thin rather than obese, elderly or a child rather than a young adult, and male rather than female. Consultation is also recommended for patients with progressive visual loss despite treatment, signs or symptoms not due to increased intracranial pressure, or any inflammatory intraocular signs that might suggest an inflammatory rather than idiopathic etiology.

## CASE FOUR (B): NEURORETINITIS: WHAT'S IN A NAME?

A 14-year-old girl presents with headache, visual loss to counting fingers vision, and severe bilateral disc edema with macular star figure OU. The outside ophthalmologist makes the diagnosis of “bilateral neuroretinitis” due to cat scratch disease and sends the Bartonella titer. There is an incomitant esotropia and the secondary diagnosis of “sensory esotropia” is made. The cat scratch titer returns 2 weeks later but is completely negative. The patient is referred to the neuro-ophthalmology service where an MRI reveals a large intracranial tumor producing papilledema OU with significant leakage of exudate into the macula bilaterally.

Bilateral optic disc edema with a macular star figure (ODEMS) is NOT by itself diagnostic for infectious neuroretinitis. The ophthalmoscopic findings are not pathognemonic and papilledema can mimic the fundus appearance of ODEMS. It is my recommendation to consider neuroimaging for all bilateral optic disc edema cases.

## CASE FIVE: BEWARE “RETINAL MIGRAINE”

A 60-year-old male judge with hypertension and diabetes describes a single episode of monocular transient visual loss OD. A dark curtain descended from superior to inferior OD, lasted 10 minutes and then spontaneously resolved. The patient was seen by an outside ophthalmologist and a diagnosis of “retinal migraine” was made. The next day the patient suffers two more episodes of similar visual loss and calls the ophthalmologist back and is given a referral for a two week appointment to neurology for “migraine”. That night the patient suffers a hemispheric stroke with hemiparesis and aphasia. A carotid study shows near occlusion of the right internal carotid artery.

The diagnosis of “retinal migraine” is one of exclusion. In elderly patients the diagnosis should be made with caution in the absence of headache history, prior attacks without residual or sequelae, and in patients who have vasculopathic risk factors. The International Headache Society (IHS) criteria for the diagnosis include: “≥ 2 attacks of “fully reversible” monocular visual loss with migraine headache”. The presumptive vasospasm mechanism for migraine headache and visual aura has fallen out of favor and spreading depression of cortical neuronal activity is a more plausible explanation for the visual symptoms in migraine. Unfortunately there is no diagnostic test for the migraine aura and a convincing history is required for the diagnosis. The role of the ophthalmologist is to exclude permanent visual loss after the event by performing formal visual field, documenting that there is no permanent residual loss, and ruling out alternative etiologies or eye markers for ischemic disease (e.g., central or branch retinal artery occlusion, Hollenhorst plaque). The most characteristic historical features for migraine are positive (e.g., scintillation or fortification scotoma) rather than negative (e.g., a black curtain) visual phenomenon; march and build up across the visual field in a nonvascular distribution, stereotyped quality with repeated events without permanent sequelae; and preferably the headache follows the aura after a few minutes duration. The most worrisome historical features for ischemia as the cause for amaurosis fugax include older aged, vasculopathic patient with first onset of an altitudinal (e.g., curtain over my vision) onset and disappearance, duration of minutes (1-10”), and seconds in onset. I would consider ordering a work up in transient monocular visual loss for patients with altitudinal history (e.g., curtain of visual loss), visible embolus, residual visual field defect, vasculopathic risk factors, and an ischemic time course. I would expedite the evaluation if the course was one of an increasing crescendo of events. On the other hand, I often defer work up for visual loss symptoms which have been present for years (e.g., hundreds of events with no residual loss) or have longer duration (e.g., 1-2 hours to days); are non-altitudinal events, the patient has no vasculopathic risk factors, or gives only a vague history of blurring. The clinician should also try to elicit a history suggestive of fluctuating rather than transient visual loss that might suggest the more common benign etiologies like ocular surface disorders or dry eyes. I will give these latter patients a pinhole to try during the events and if it alleviates the problem then it can be safely assumed to be ocular surface or refractive in nature. I also ask the patients to keep a diary of their events to establish some of the historical details above and I often give an empiric artificial tear trial.

## SUMMARY

There are neuro-ophthalmic afferent system diagnoses that a general ophthalmologist should (almost) never make alone because they are dangerous and could be due to an underlying vision or life threatening etiology. Consultation with colleagues in neurology or neuro-ophthalmology is recommended in these settings including: 1) unexplained optic atrophy, 2) posterior ischemic optic neuropathy, 3) chronic optic neuritis, 4) papilledema, and 5) ocular migraine in elderly patients.
